# Four-phase rhinomanometry: a multicentric retrospective analysis of 36,563 clinical measurements

**DOI:** 10.1007/s00405-015-3723-5

**Published:** 2015-07-22

**Authors:** Klaus Vogt, Klaus-Dieter Wernecke, Hans Behrbohm, Wolfgang Gubisch, Mara Argale

**Affiliations:** Schwarzer Weg 3 B, 18292 Krakow Am See, Germany; Faculty of Medicine, Centre of Experimental Surgery, University of Latvia, Riga, Latvia; Sostana GmbH, Wildensteiner Strasse 27, 10318 Berlin, Germany; PARK-KLINIK Weissensee GmbH, Schönstrasse 80, 13086 Berlin, Germany; Marienhospital Stuttgart, Böheimstrasse 37, 70199 Stuttgart, Germany

**Keywords:** Four-phase rhinomanometry, Meta-analysis, Parameter, Effective resistance, Logarithmic transformation, Clinical validation

## Abstract

Rhinomanometry can still be considered as the standard technique for the objective assessment of the ventilatory function of the nose. Reliable technical requirements are given by fast digital sensors and modern information technology. However, the xyimaging of the pressure-flow relation typically shows loops as a sign of hysteresis, with the need for resolution of the breath in four phases. The three pillars of 4-phase rhinomanometry (4PR) are the replacement of estimations by measurements, the introduction of parameters related to the subjective sensing of obstruction, and the graphical information regarding the disturbed function of the nasal valve. In a meta-analysis of 36,563 clinical measurements, we analyze the errors of the “classic” parameters (flow in 150 Pa) and reject the further use of these parameters as obsolete, because they correspond to an inaccurate estimation rather than proper measurement. In a pre-study of 1580 measurements, the logarithmic effective resistance (Reff) was found to have the highest correlation with values obtained from a visual analog scale. Next, we classify the inspiratory effective resistance in 20,069 measurements without treatment and 16,494 measurements after decongestion with xylometazoline 0.1 % spray in 20 % percentiles. The gradation of obstruction delivers not only “normal” values but also indications for the severity of the obstruction in adult Caucasian noses. Adoption of the distribution for the growing nose and analysis of the total nasal resistance is addressed, and typical findings of nasal valve phenomena are outlined.

## Introduction

The introduction of computer-aided rhinomanometry and the replacement of previous graphic methods around 1980 [1–4] can be considered a milestone in the functional diagnostics of the nasal air stream. Personal computers appropriated the method in daily practice. At the same time the recorded xy curves showed repeated loops instead of the expected simple lines, which have since been in part identified as technical errors due to different compartments of the system, in particular different speed and sensitivity of the used transducers. From 1992 on, rapid sensors eliminated these errors, and today highly sensitive and fast digital sensors for pressure and mass flow represent the state of the art.

In 1994, during the conference of the European Rhinologic Society in Copenhagen, Vogt and Hoffrichter [5] proposed the term “high-resolution rhinomanometry” for a procedure resolving the entire breathing cycle into four phases: the accelerating inspiratory phase, the decelerating inspiratory phase, the accelerating expiratory phase, and the decelerating expiratory phase. This discrimination became necessary because errors arising from the technical equipment had previously been systematically excluded. In subsequent years, countless model experiments as well as the simulation of nasal breathing by computational fluid dynamics (CFD) confirmed four-phase rhinomanometry (4PR) as a theoretically and technically well-founded diagnostic method for the physiologic investigation of the nasal air stream. The state of the art in 2009 was summarized in Supplement 21 of the journal Rhinology by 12 members of an international and interdisciplinary consortium [6]. 4PR is now used in more than 20 countries in clinical rhinology, plastic surgery, and sleep medicine. This meta-analysis is the first presentation of comprehensive clinical material.

The three pillars of four-phase-rhinomanometry are: the replacement of estimations by measurements, the introduction of parameters related to the subjective sensing of obstruction, and the graphical information regarding the disturbed function of the nasal valve.

Given the ongoing discussion about the differences and the clinical usefulness of “classic” rhinomanometry and 4PR, the following issues of clinical interest and high importance in experimental studies about the respiratory function of the nose have been investigated:

- The diagnostic power and accuracy in “classic” rhinomanometry of measured flow at a differential pressure of 150 Pa and its incorrect derivation “Resistance at 150 Pa”
- The distribution of the parameters effective resistance and vertex resistance and their logarithmic derivations within a population of healthy and diseased noses before and after decongestion by xylometazoline, the subsequent classification of clinical results, and their correlation between sensation and objective obstruction.

## Materials and methods

The rhinomanometric databases of five different German ENT hospitals that have been using 4PR for more than 5 years are analyzed in this study. Three departments are dealing with general otorhinolaryngology and two hospitals are specialized in facial-plastic surgery. The age range of patients was 14–82 years. In 20,069 untreated nasal sides, active anterior rhinomanometry was carried out. A total of 16,494 measurements were subsequently followed by a decongestion test with xylometazoline 0.1 % spray and a second measurement 10 min later.

All protocols were reviewed with regard to technical errors. A total of 157 measurements obtained from non-Caucasian noses or children were excluded from the study. All measurements were carried out using the 4PR rhinomanometer models HRR3 or 4RHINO (Rhinolab, Freiburg, Germany) with software version 3.57, 4.31, or 5.01. The software of this system is Windows-based and the format of the data stored in the databases has been identical since 1999. The following details are important for providing exact measurements and reproducible results.

1. The calibration of the device was controlled over predetermined distances; the calibration of all instruments was correct before the beginning and after the end of the studies.
2. For the coupling of the pressure tube to the nose, the “tape method” was exclusively applied. The use of any prefabricated coupling element is forbidden in the participating departments. The elastic tape Microfoam (3 M) was used. Anesthesiologic masks of different sizes (Ambu, Ballerup, Germany) were chosen. The extranasal “dead space” did not exceed 0.15 L including connection pieces and filter housing.
3. All measurements were carried out after adaptation of the patient to room temperature, at rest and in an upright sitting position.

The measurement results are stored as an average of 3–5 breathing cycles with 2000 data for flow and differential pressure according to the recommendations of the ISOANA 1984. The averaging procedure by splining was described previously by Vogt and Wernecke [2, 5]. By an export function of the 4PR program measurement, results can be directly transferred to text files for further processing with standard statistical programs. SPSS 22 and Excel 2010 with XL-Stat were used in this study for the following statistical evaluation.

Evaluated parameters

1. The nasal flow at 150 Pa differential pressure during the four phases of the nasal breathing cycle is marked as intersection points in Fig. 1. The point marked by “!” is the only point used as diagnostic information in classic rhinomanometry, a remnant of the graphical evaluation used before the introduction of computerized rhinomanometry after 1983. Prior to then, an evaluation of all information on the curve was not possible by graphical methodology.

**Fig. 1:**
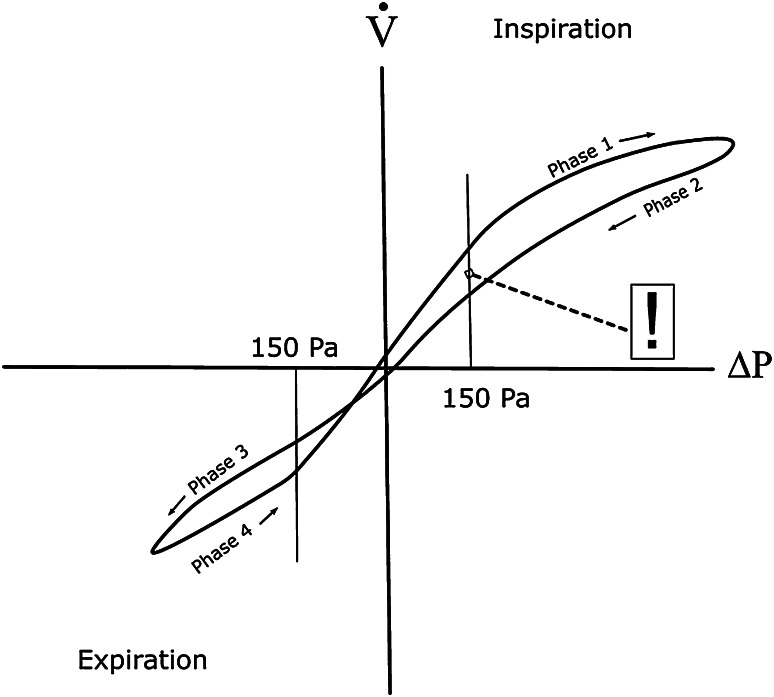
XY diagram in four-phase rhinomanometry
2. Vertex resistance (VR) (Fig. 2) and logarithmic vertex resistance (LVR) in inspiration and expiration. The VR is the linear quotient between differential pressure and flow at the highest point of the nasal air flow. VR is related to the peak flow resistance in pneumologic function tests, which is determined at the maximum of the inspiratory flow, but VR in 4PR is measured during normal quiet breathing activity. At this point of a breathing cycle the air stream is steady by definition and, because the influence of acceleration and deceleration is missing, resistance is defined by the linear relation $R = \varDelta P/\dot{V}$. The vertex of the curve is the only point where this linear relation is mathematically correct. By contrast, the application of “resistance at 150 Pa”, a parameter still used by some researchers [7, 8], must be strongly rejected as a physically and mathematically incorrect and, therefore, non-acceptable calculation in an unsteady accelerating or decelerating air stream.

**Fig. 2:**
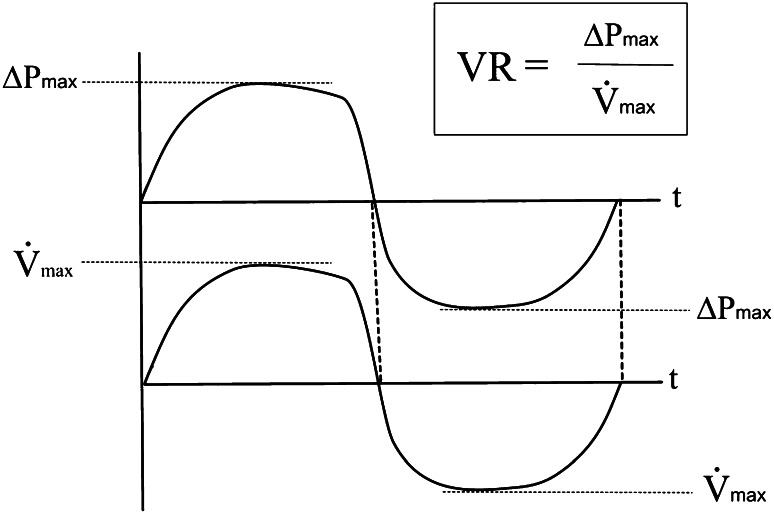
Vertex resistance
3. Effective resistance (Reff) (Fig. 3) and logarithmic effective resistance (LER) in inspiration, expiration, and for the entire breath cycle. The Reff, used for a long time in electrical engineering, is simply calculable by any computer by summing up all measurements within a given time, which corresponds with the calculation of the integral under the pressure and flow curves. In the HRR-program versions used in this study, Reff was calculated after averaging 3–5 breathing curves. The information can be obtained for the inspiratory or expiratory phase or for the entire breath. Reff is, as is VR, a measured parameter, which is representative of the energy of the entire breath; it replaces rough estimations and insufficient conclusions following one measured point, which is, in addition, not always measurable (see below).

**Fig. 3:**
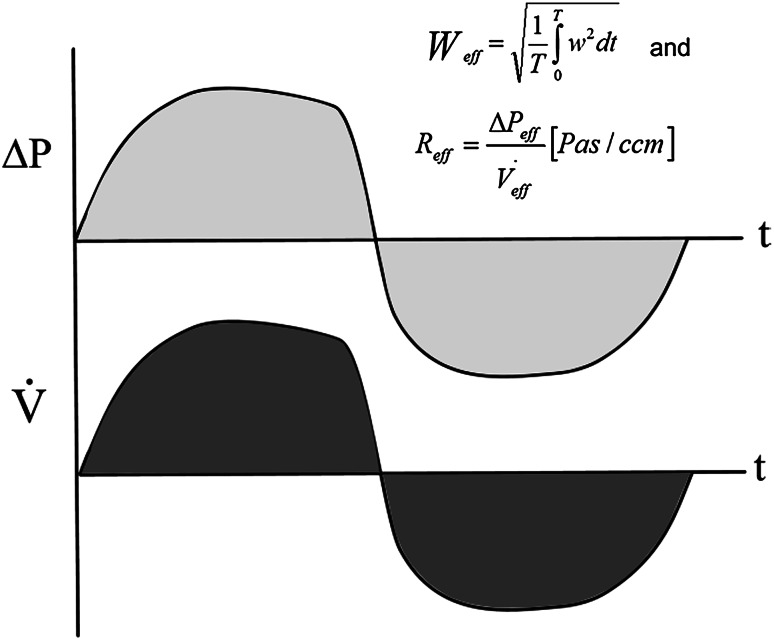
Effective resistance

The descriptive statistics of the non-classified material and the after classification are summarized in Table 1.

**Table 1:**
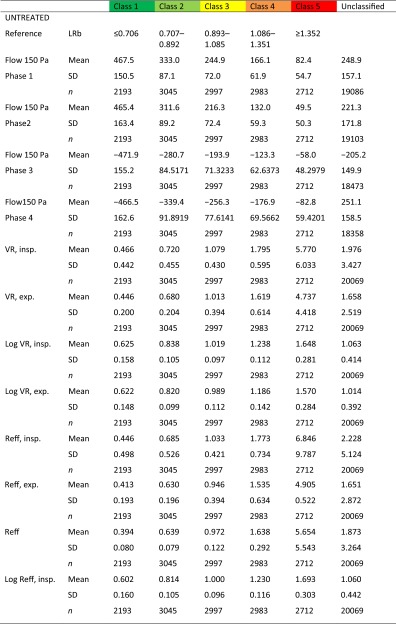

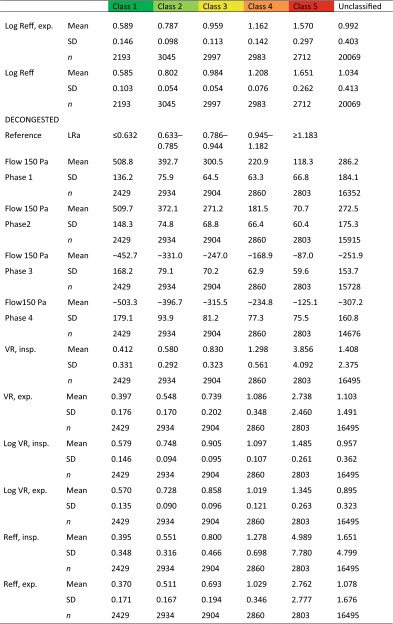

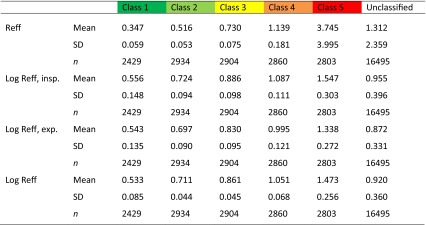
Descriptive statistics of 36,563 classified and non-classified measurements

The classes are corresponding to 20 % percentiles of the population (see Fig. 9a, b)

*exp.* expiration, *insp.* inspiration, *Reff* effective resistance, *SD* standard deviation, *VR* vertex resistance

## Part 1: analysis of the differences in nasal air flow at a differential pressure of 150 Pa in four phases of the nasal breathing cycle and their relations to the curve hysteresis

In this part of the study, 20,069 measurements before any treatment and 16,494 measurements 10 min after decongestion with 0.1 % xylometazoline spray were included. As mentioned frequently in earlier publications, some patients cannot reach the pressure level of 150 Pa or higher [6]. In this study, the number of lost observations can be read from Table 1. It should be mentioned that the use of pressure levels at 75 Pa instead of 150 Pa, as is sometimes practiced, is already in a critical region of the nasal air flow, where the unsteady flow is maximally accelerating and the influence of noise is high. A pressure level of 75 Pa is equal to 7.5 % of the full signal output. Using this parameter is as incorrect as it is unnecessary. Under the condition of quiet breathing in a resting state, the data loss in flow measurements at 150 Pa was found as displayed in Table 2.

**Table 2:** Lost results because of non-reached pressure level of 150 Pa

	Total	Phase 1	Phase 2	Phase 3	Phase 4
Before decongestion	20,069	19,275	19,103	18,571	18,482
No result		894	966	1498	1587
No result (%)		4.45	5.04	7.84	8.55
After decongestion		15,962	15,918	15,511	15,471
No result		532	576	983	1023
No result (%)		3.23	3.61	6.18	6.60

**Fig. 4:**
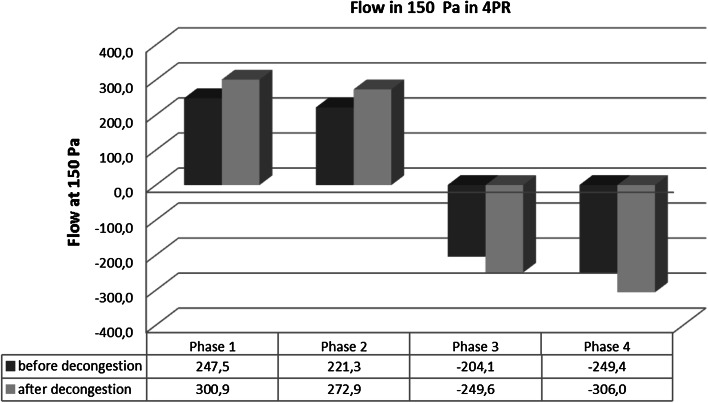
Statistical differences for the flow at 150 Pa in four breathing phases

Within the remaining cohort the means were calculated for the flow in 150 Pa (Fig. 4).

The order phase 1 > phase 2 > phase 3 < phase 4 is clearly visible and appears in the same order in all subgroups investigated previously. All differences are significant at a probability level *p* < 0.0001. The descriptive statistics of the flow differences between phases 1 and 2 and between phases 3 and 4 (in percent) are shown in Table 3.

**Table 3:** Descriptive statistics of the differences between phases 1 and 2 and phases 3 and 4

	Difference in flow 150 (%)			
	Before decongestion		After decongestion	
	Phase 1–2	Phase 3–4	Phase 1–2	Phase 3–4
Arithmetic mean	15.6	−30.0	13.4	−28.7
Median	9.1	−17.2	7.2	−17.3
Standard deviation	22.5	38.0	20.2	32.6
Skewness	1.2	−1.9	1.4	−1.9
Minimum	−31.5	−217.9	−26.6	−179.4
Maximum	99.2	34.4	91.4	15.5
Number	18,129	17,489	15,118	14,605

**Fig. 5:**
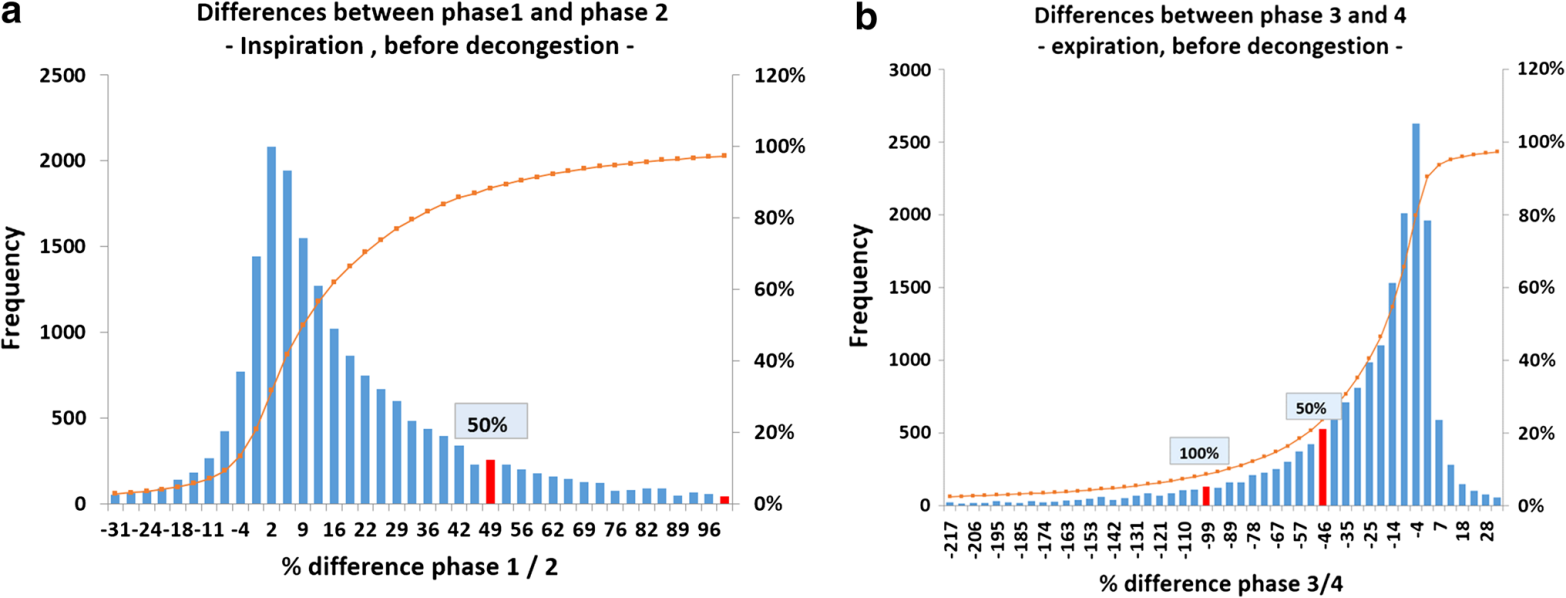
Histograms of the differences for the flow at 150 Pa between different breathing phases (%)

The histograms of the differences between phases 1–2 and 3–4 (Fig. 5) imply the following clinically important conclusions. In objective measurements, where the activation of the nasal valve was not provoked, during inspiration a difference of 100 % of the higher value between phase 1 and phase 2 was observed in 45 cases, and a difference of 50 % in 1993 cases.

In summary, the statistical facts presented here, in addition to numerous single observations in typical pathologic cases, confirm the necessity of resolution of the nasal breathing curve in four phases as being of clinical importance.

**Fig. 6:**
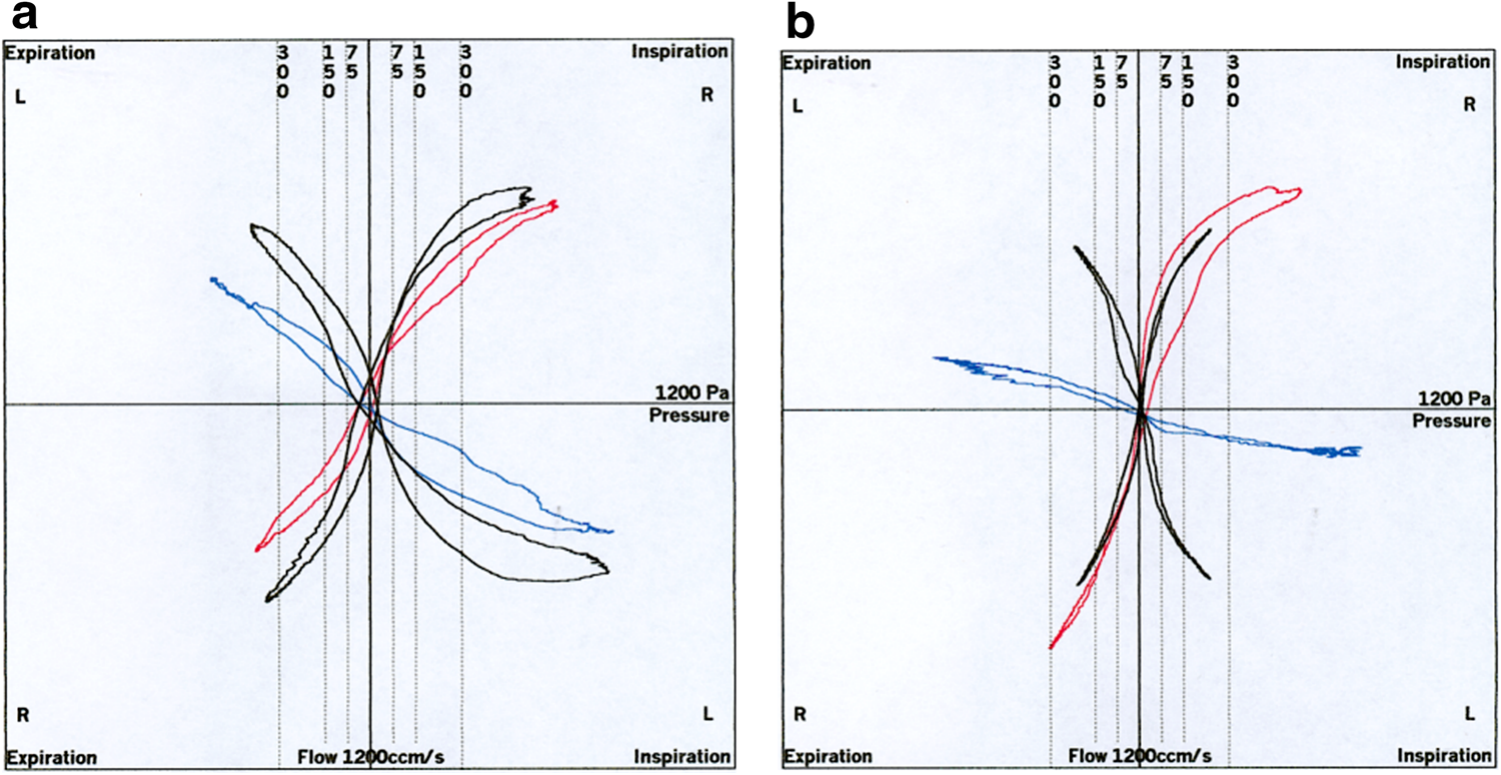
**a**, **b** Typical “valve phenomena” in four-phase rhinomanometry

Besides the mandatory numeric information, the visual information is also missing, which is obtained when the elastic properties and Bernoulli phenomena release the so-called valve effects, leading the surgeon to a more precise indication for surgical intervention to improve the nasal obstruction [6]. Two typical examples are shown in Fig. 6a, b. In the case of Fig. 6b the onset of the valve activity starts only in elevated flow and acceleration, while during “quiet” breathing the onset of the aspiration of the nasal wing could not be observed.

The observed differences between phases 1/2 and 3/4 show clearly that the hysteresis of the curves, which can be seen only by 4PR, is not a negligible physiologic phenomenon. The experiments of Gross and Peters [9] showed clearly the influence of the volume and the “storage effect” of large volumes in rigid models. In all measurements of this meta-analysis the moved volume outside the nasal cavity, i.e., the volume of the mask and connective parts, did not exceed 0.150 L. Under such conditions, a visible hysteresis can be generated by the system only under abnormal breathing conditions. In this case, the hysteresis is symmetric and the curves are running through the intersection of the flow and pressure axis. Rigid models, as used also by the authors [10], can show in addition that there is a symmetric hysteresis around the xy intersection if the nose is approaching the shape of a tube rather than a bore (“diaphragm”). It is of highest clinical importance that the passive movement of the elastic structures of the nasal entrance produces a large asymmetric hysteresis. This hysteresis is released by a dynamic reduction of the cross-sectional area of the “valve region” by Bernoulli effects, depending also on the elastic tissue properties arising from the acceleration of the flow during phase 1. The resistance during phase 2 is then elevated by the air flow generated in the first phase. The motility of the nasal entrance is a normal physiologic phenomenon, corresponding to a “shock absorber” against rapid inhalation of unconditioned air or generation of more turbulence in “sniffing.” If the rhinomanometric loop is not running through the axis intersection, the nasal valve is working in a manner comparable to a hornpipe: the flow is still running without any additional pressure supply. If these phenomena are observed under “normal” breathing conditions, the ENT surgeon has to direct his activities to the nasal valve as well. Because of the different compartments contributing to the generation of hysteresis, it would seem inadvisable to correct the “rigid part” mathematically. It is easy to read from the graphical result whether hysteresis in a record is of clinical importance.

In summary and in conclusion of this part of our analysis, we reject strongly the statement and the conclusions of Wong and Eccles [8], deduced from primitive and inadequate experiments in rigid models, which did not show differences between the breathing phases. “Simplicity” claimed in medical diagnostics is a quantitative term, defined by the relationship between technical feasibility and the skills and intelligence of the user. If “simple” methods implicate false conclusions, they must be excluded from the diagnostic inventory.

## Part 2: the classification and clinical meaning of the parameters effective resistance and vertex resistance

In the literature over many years, in theoretical considerations of the nasal air stream, the terms “laminar” and “turbulent” air stream are found, as well as non-successful attempts to find a mathematical equation describing the relation between pressure and flow of the nasal air stream. Under the auspices of contemporary research in fluid dynamics, we know that:

- The nasal air stream is to a great extent an “unsteady” air stream by definition, which is permanently and quickly changing its velocity and direction within an irregular structure. It is always in part turbulent and laminar, which can be easily demonstrated by computational fluid dynamics.
- If the shape of the nasal channel corresponds to a “diaphragm” or “bore” or “hole,” the air flow is preferably turbulent; if the shape of a nearly completely obstructed nose is more similar to a tube, where the length exceeds 20 times the diameter, the air stream becomes laminar.
- Bernoulli effects play an important role in affecting the desired closure of the nose while sniffing or sucking the nasal mucus backwards. These effects are not reproducible and depend also on minor variations of the nasal anatomy, the elastic properties of the nasal wall, and the acceleration of the inspiratory air flow. They are also present at the conscious release of the closure.

Against this background, parameters describing the nasal air stream should have the following properties:

1. Parameters should describe the relation between pressure and flow or, furthermore, the energetics of nasal breathing, without any regard to the shape of the nasal breathing wave.
2. Parameters must be measurable in any case. Estimations have to be replaced by measurements whenever possible.
3. Parameters should be statistically related to the sensing of obstruction, i.e., the subjective feeling of the patient.

**Fig. 7:**
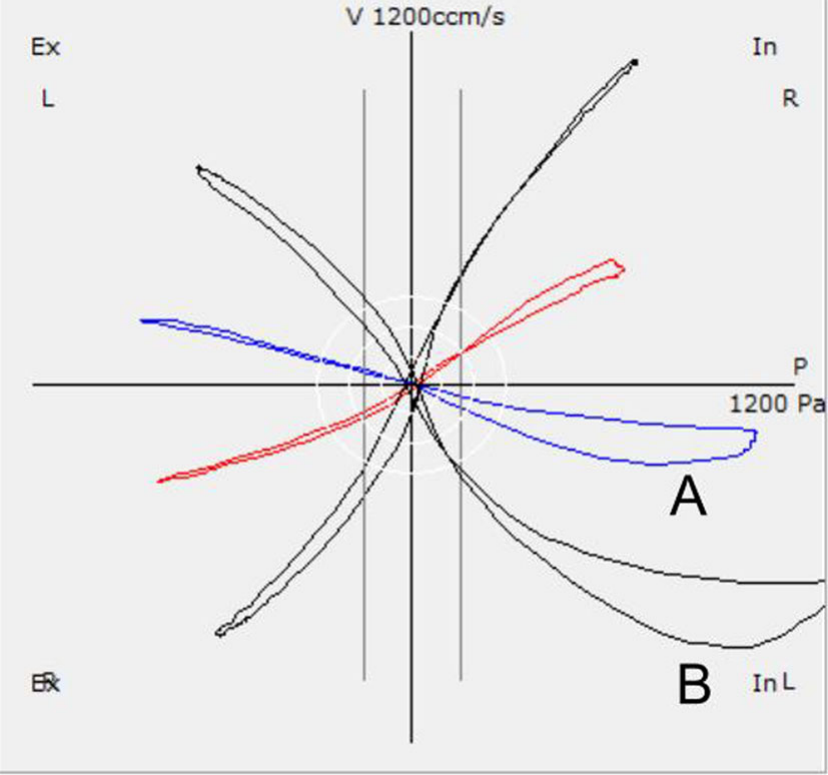
Typical valve phenomena. In curve *A*, Log VRin = 1.45 and Log Reffin = 1.69; in curve *B*, Log VRin = 1.06 and Log Reffin = 1.14 Pa/cm^3^

These criteria are met for the VR as the only acceptable point-measurement within the breathing cycle and for the Reff, which is in fact the summation of a series of rapid measurements in a millisecond distance, computed for the inspiratory or expiratory part or for the entire breath. The resistance as a relation between pressure and flow is no longer calculated by a division of points but by a “division of areas”. VR and Reff are highly correlated to each other and show only very low differences in a pooled cluster. However, they do differ when the ascending nasal air stream releases the Bernoulli effect at the nasal valve and the air channel is narrowing. In this case, the resistance during the descending inspiratory part (phase 2) is higher, and the Reff exceeds the value of the VR (Fig. 7).

There are two reasons to use VR and Reff as diagnostic parameters after logarithmic transformation:

1. It was shown as early as 2003 [11] that the statistical distribution in histograms of VR and Reff approaches a normal distribution after logarithmic transformation, which allows a classification of the obstruction in an easier way.
2. Every sensory message, the sensation of force or power, which is necessary for the work of nasal breathing, follows the well-known logarithmic scale of Weber–Fechner, which states that subjective sensation is proportional to the logarithm of the stimulus intensity. Relating the measured degree of nasal obstruction to the sensation of the work of breathing as given on a logarithmic visual analog scale (VAS) is much more informative after using logarithmic scales for both parameters (see below).

Under these preconditions, we proposed a classification for nasal obstruction using logarithmic parameters after multiplication of the measured value by 10, which was proposed as more practicable in daily clinical work [6] through avoiding negative numbers for the resistances. The abbreviations in Table 4 were chosen.

**Table 4:** Abbreviations for 4PR parameters

	Value	Logarithmic transformation [Log(10 * value)]
Vertex resistance, inspiration	VRin	LVRin
Vertex resistance, expiration	VRex	LVRex
Effective resistance, inspiration	Reffin	LReffin
Effective resistance, expiration	Reffex	LReffex
Effective resistance, total breath	Reff	LReff

In a preceding study, the same parameters applied in this meta-analysis were correlated with results obtained from a VAS scale, which has been implemented in the applied software for 15 years. The values are generated by shifting a “button” from the middle to the right or left side based on the actual feel of obstruction. In the case of a repeated measurement after application of nasal spray, the patient adjusts the button after the second measurement.

In this study, a classification of the subjective values was obtained by setting up 20 % percentiles of the population (Table 5).

**Table 5:** Classification of subjective obstruction following a visual analog scale (see Fig. 8a, b)

Percentiles	Class	Before decongestion	After decongestion
0–19	1	≤14	≤59
20–39	2	15–30	60–71
40–59	3	31–61	72–79
60–79	4	62–66	80–88
80–100	5	>66	>88

**Fig. 8:**
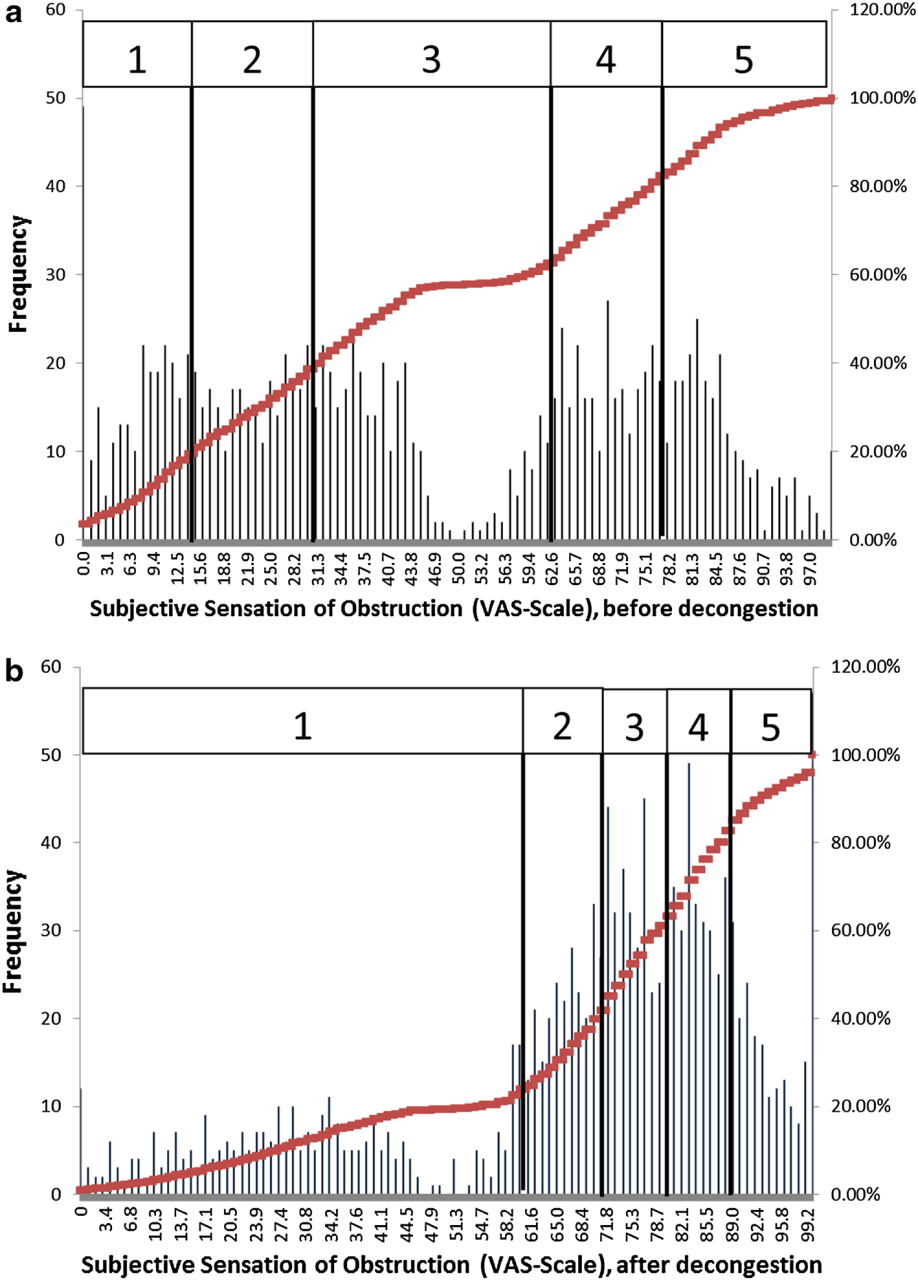
**a**, **b** Histograms of subjective obstruction measured on a visual analog scale

The histograms shown in Fig. 8 show a steady distribution of frequencies, while the “gap” in the middle is regarded as the effect of the start point of movement of the “button” at 50 units. The second histogram shows a dramatic change after the application of xylometazoline, when the number of “good noses” increases.

The correlation analysis (cf. Pearson’s correlation) of the data yields the results from one subjective and various objective parameters (Table 6). All of the correlation coefficients are highly significant (*p* < 0.0001).

**Table 6:** Correlation between subjective obstruction based on a visual analog scale and 4PR parameters

	Before decongestion	After decongestion
Flow 150 phase 1	0.495	0.469
Flow 150 phase 2	0.500	0.443
Flow 150 phase 3	−0.495	−0.505
Flow 150 phase 4	−0.508	−0.509
VR, inspiration	−0.391	−0.332
VR, expiration	−0.318	−0.450
Log VR, inspiration	−0.543	−0.529
Log VR, expiration	−0.529	−0.513
Reff, inspiration	−0.377	−0.315
Reff, expiration	−0.367	−0.344
Reff	−0.387	−0.323
Log Reff, inspiration	−0.549	−0.535
Log Reff, expiration	−0.530	−0.521
Log Reff	−0.553	−0.546

**Fig. 9:**
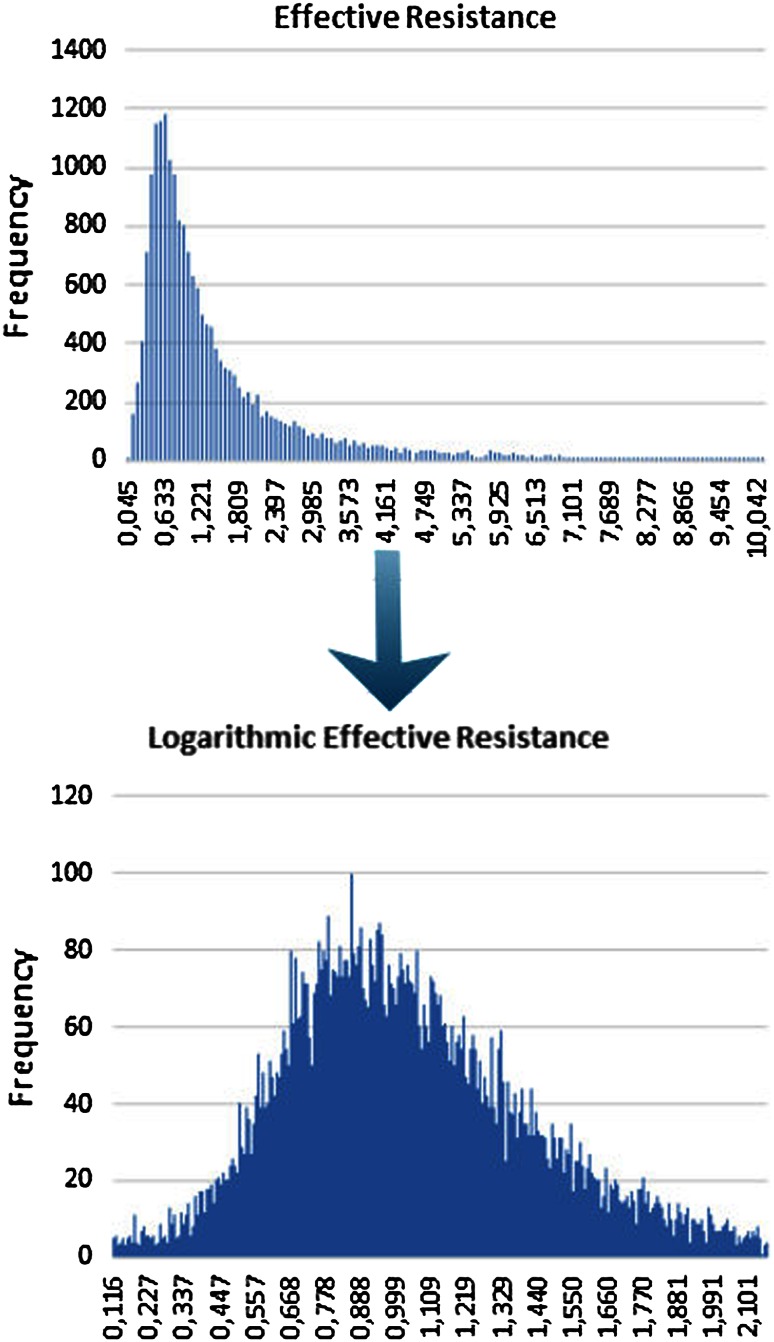
Effect of logarithmic transformation on the statistical distribution of effective resistance values

A comparison of the statistical distribution of the values before and after logarithmic transformation (Fig. 9) shows clearly that a normal distribution can be only achieved for logarithmic values. It follows that only the logarithmic values can be related to the distribution of the subjective values as obtained from the VAS scale, implicating a statistically significant correlation.

From Table 6, it could be stated that the highest correlation between objective parameters and subjective sensation can be achieved with the LER, and that there are only small statistical differences in the correlation between inspiration and expiration as well as with LVR. The corresponding classification based on 20 % percentiles for the highest correlated LER with these data resulted in the situation as shown in Table 7.

**Table 7:**
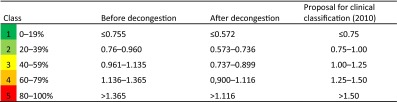
Classification of logarithmic effective resistance (pre-study) (*n* = 1580) [6]

**Fig. 10:**
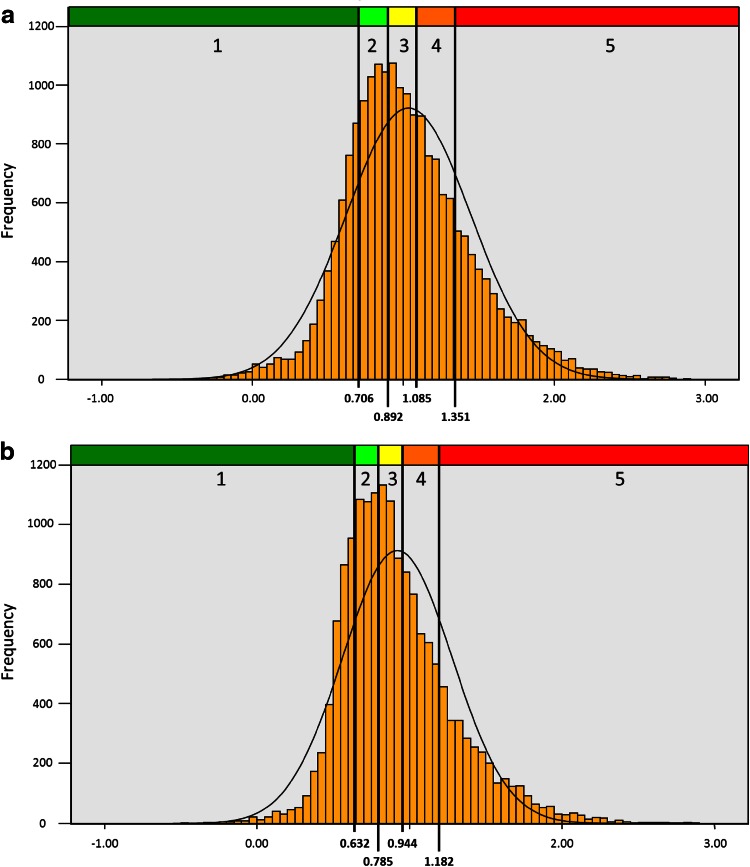
**a**, **b** Histograms of the statistical distribution of Log Reffin before and after decongestion

The clinical results of 20,069 untreated nasal sides and 16,494 measurements after a decongestion test with xylometazoline based on 20 % percentiles for the LER in inspiration confirmed these findings (Table 8) and are represented in the histograms of Fig. 10.

**Table 8:**
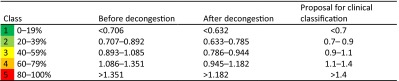
Classification of logarithmic effective resistance (*n* = 36,563)

The proposal for the clinical classification is also valid for the Logarithmic effective resistance when measured only in inspiration or expiration and the logarithmic vertex resistance in inspiration and expiration

## Discussion

The International Standardization Committee for the Objective Assessment of the Upper Airway was founded in Brussels in 1984. It published two important recommendations [11, 12] in the initial standard of 1984, which was based on the existing graphical methods at the time and the recommendations following a consensus conference in Brussels in 2003 [13]. After the introduction of high-resolution rhinomanometry, later to become 4PR, the attitude of the ISOANA toward the new method was critical because of suspected technical errors. By countless model experiments, simulation by CFD and, again, the model experiments of Gross and Peters [9], it has been proven that the hysteresis causing the loops in rhinomanometric curves is a phenomenon that can appear as a result of certain anatomic structures depending on speed and acceleration of the air flow. In addition, the influence of the elasticity and the release of Bernoulli effects may create typical curves of clinical importance.

Accepting these facts, the causes of differences in flow measurements between the breathing phases are explainable, and the statistics herein show the extent of false or missing data. In addition, the calculation of linear resistance by the flow values at a single point is physical and mathematical nonsense, and the information about the flow at a given pressure delivers only an estimation of limited value for the critical analysis of nasal breathing, which may be in many cases erroneous. The calculation of the total nasal resistance can be better carried out with the correct parameters VR and Reff. The recent clinical review by Clement et al. [14] refers only to the state of the art in experimental and clinical research before the consensus conference in Brussels in 2003 and is not based on clinical data. Recent publications have not been considered by these investigators. It was initially a merit of the ISOANA to introduce SI units and to contribute to the mutual understanding of rhinologists worldwide, but under the auspices of quality management and correctness of the diagnosis, obsolete methods as described herein should no longer be recommended. Any quality management of medical devices must exclude methods based on false calculations or estimations if exact measurements can replace them.

The relation between objective measurements and the feeling of obstruction has been repeatedly discussed during recent years. Nearly all publications refer only to classic rhinomanometry and have found no or only weak correlations [15–17]. The fact that logarithmic values lead to a statistically significant correlation has not been published sufficiently to date given its requisite clinical importance; it can be found only in Supplement 21 of journal Rhinology [6]. The introduction of Reff and its logarithmic derivation is a means to be independent from the pressure-flow relation at a given point and invalid calculations of resistances within an unsteady air stream. For the work of breathing, the shape of the breathing curve is less important than the power necessary to maintain breathing throughout the entire breath. The additional information provided by VR is important for the numeric description of the valve effect in curves with expressed hysteresis due to valve phenomena.

Recommendations for the clinical classification of rhinomanometric measurements have been given previously by Bachmann [18] and Vogt [19] for the graphical and first computer-assisted rhinomanometric records, but these are estimations following the clinical experience of the authors. The classification presented herein of a comprehensive concept is based on the distribution of 36,563 measurements in 20 % percentiles, whereas a simplified clinical classification as formerly proposed may be a matter of discussion.

## Conclusions

The parameters Reff and VR and their logarithmic derivations have been proved to be capable of measuring the degree of obstruction of the nasal airway. The classification, which as of now is valid for adult Caucasian noses, will be adapted for other races and in a dynamic way for the growing nose in childhood. The investigated parameters can be implemented in any system of computerized rhinomanometry. This approach should replace inaccurate methods of estimation as used in so-called classic rhinomanometry and be exclusively applied in pharmacologic clinical studies. In a subsequent report, we will analyze the connotations of 4PR for measurements of total nasal resistance.
